# Quantitative Comparison of UAS-Borne LiDAR Systems for High-Resolution Forested Wetland Mapping

**DOI:** 10.3390/s20164453

**Published:** 2020-08-10

**Authors:** Narcisa Gabriela Pricope, Joanne Nancie Halls, Kerry Lynn Mapes, Joseph Britton Baxley, James JyunYueh Wu

**Affiliations:** Department of Earth and Ocean Sciences, University of North Carolina Wilmington, 601 S. College Rd., Wilmington, NC 28403, USA; hallsj@uncw.edu (J.N.H.); mapesk@uncw.edu (K.L.M.); jbb8509@uncw.edu (J.B.B.); jjw7387@uncw.edu (J.J.W.)

**Keywords:** Unmanned aerial systems, UAS, LiDAR, wetlands delineation, mapping, topographic modeling

## Abstract

Wetlands provide critical ecosystem services across a range of environmental gradients and are at heightened risk of degradation from anthropogenic pressures and continued development, especially in coastal regions. There is a growing need for high-resolution (spatially and temporally) habitat identification and precise delineation of wetlands across a variety of stakeholder groups, including wetlands loss mitigation programs. Traditional wetland delineations are costly, time-intensive and can physically degrade the systems that are being surveyed, while aerial surveys are relatively fast and relatively unobtrusive. To assess the efficacy and feasibility of using two variable-cost LiDAR sensors mounted on a commercial hexacopter unmanned aerial system (UAS) in deriving high resolution topography, we conducted nearly concomitant flights over a site located in the Atlantic Coastal plain that contains a mix of palustrine forested wetlands, upland coniferous forest, upland grass and bare ground/dirt roads. We compared point clouds and derived topographic metrics acquired using the Quanergy M8 and the Velodyne HDL-32E LiDAR sensors with airborne LiDAR and results showed that the less expensive and lighter payload sensor outperforms the more expensive one in deriving high resolution, high accuracy ground elevation measurements under a range of canopy cover densities and for metrics of point cloud density and digital terrain computed both globally and locally using variable size tessellations. The mean point cloud density was not significantly different between wetland and non-wetland areas, but the two sensors were significantly different by wetland/non-wetland type. Ultra-high-resolution LiDAR-derived topography models can fill evolving wetlands mapping needs and increase accuracy and efficiency of detection and prediction of sensitive wetland ecosystems, especially for heavily forested coastal wetland systems.

## 1. Introduction

### 1.1. A Brief History of Wetlands Mapping in the United States

Wetland identification and delineation is conventionally a time-consuming, laborious process involving ground-based assessment of the region’s hydrology, soil type, and plant species identification. In the United States, the Corps of Engineers (COE) is responsible for mapping wetlands per the 1972 Clean Water Act, Section 404, regulatory program and have therefore developed comprehensive mapping guidelines still largely in use today [1]. Additionally, the US Fish and Wildlife Service (FWS) has developed a detailed wetland mapping protocol [2] based on the standard Cowardin wetland classification scheme [3]. The distinction between the COE and FWS is that the COE guidelines are for the sole purpose of mapping a wetland boundary versus the FWS protocol is for classifying the many types of wetlands. Therefore, the COE approach identifies where a wetland is located and the FWS goes further by not only identifying the location of a wetland, but also providing detailed information about the type of wetland. It is important to recognize that the COE approach is mandated by legal statute and therefore all proposed land development must adhere to these guidelines, but the FWS approach, which is more detailed from a land cover perspective, is not required for any land to be developed (e.g., to obtain a building permit), nor are the COE data publicly available. Conversely, the FWS publishes the National Wetlands Inventory (NWI) data but given that it is not mandated by legal statute, these data are not regularly updated. The definition of what is considered a wetland also differs between the COE and FWS. When deciding if an area is a wetland, the COE requires that all three parameters (vegetation, soil type, and hydrology) are met while the FWS only requires one of the three to meet the guidelines for a wetland. The two agencies do rely on the same plant species list as designated by the FWS system. To make matters more complicated, each state must adhere to federal guidelines of the COE because of the mandate from the Clean Water Act, but they can also adopt additional rules for defining wetlands. For example, in North Carolina, the Wetland Assessment Method (NC WAM) was developed in 2015 for the purposes of collecting accurate and useful wetland information to be used for planning and assessing wetland function [4,5]. Unfortunately, this detailed wetland information is collected for a single point location, with no guidelines for mapping the wetland itself because the assumption is that the wetland has already been mapped by the COE. However, there is no spatial or geographic database of mapped COE wetlands since each is mapped for individual projects and are not integrated into a state or national database of mapped wetlands. Therefore, the available mapped NWI wetlands are older and mapped to 1:24,000 scale, while the current and higher spatial resolution wetlands mapped through the required COE permitting process are not available. Because of these shortcomings, researchers have investigated alternative approaches to mapping wetlands for many years from historical aerial photography, satellite imagery, and more recently through Unmanned Aerial Systems (UAS).

Historically, many satellite remote sensing platforms and image processing techniques have been developed and tested for mapping wetlands [6,7,8,9,10]. Although many wetland characteristics may be evaluated using remote sensing, the limited spatial and temporal resolutions of satellite imagery make it difficult to examine wetlands in the detail required to classify and delineate them to the mapping standards required by the Corps of Engineers. Forested coastal wetlands are especially difficult to map because of the dense forest canopy unlike other wetlands (e.g., non-forest/grass wetlands); when they occur in tidal areas, they are even more problematic because of the tides. Given the numerous challenges posed by mapping the wide variety of coastal wetlands, and the particular difficulties of resolving terrain under thickly forested coastal wetlands, this paper focuses on demonstrating the efficacy of UAS technologies to overcome challenges of remote sensing of forested wetlands. UAS technology allows similar data to be collected, but at significantly higher spatial and temporal resolutions, allowing for more accurate mapping and refined analyses.

### 1.2. Use of Unmanned Aerial Systems (UASs) in Wetlands Mapping

The two main sensor types associated with UAS-based wetland analysis are multispectral and light detection and ranging (LiDAR) systems. The former has proven highly effective for vegetation classification as well as for evaluating soil moisture content [11]. The use of LiDAR for mapping wetlands is fairly new, but due to the ability of LiDAR to collect elevation data from beneath the vegetation canopy and specialization in detecting topographic features, LiDAR produces high quality digital surface models (DSMs) and is ideal for evaluating hydrologic features [12]. Prior studies have explored applications of this technology as a stand-alone data-type for flood risk evaluations [13]; however, there is little documentation of its application in effective wetland classification when combined with multispectral sensors. Given LiDAR’s efficacy in mapping hydrologic features and multispectral data’s value in evaluating plant communities and soil moisture, analyses that use a combination of the two data types may provide a more accurate approach for identifying and mapping wetlands. In this paper, building towards the goal of enhanced wetland classifications, we show the relative accuracies of two variable-cost LiDAR sensors ($80,000 to $175,000) mounted on the same platform in revealing high resolution wetland topography.

The best-known producer of multiple spinning laser scanners currently on the market is Velodyne, with the VLP-16—referred to as the Puck—and the HDL-32E sensors [14]. There is also the lightweight version of the Puck—the Puck LITE—which has identical advertised performance but weighs 300 g less [14]. Both the Puck and the Puck LITE have been used on UASs in forestry studies; Liu et al. (2018) used a UAS with the Puck and partial least squares (PLS) modeling to estimate plot-level planted Ginkgo forest structural attributes (e.g., diameter at breast height [DBH], Lorey’s mean height, etc.) with better accuracy than previous airborne laser scanning (ALS) estimation methods [15]. They attributed this to the UAS-LiDAR’s lower flight altitude and slower flight speed, which allowed greater average point density than that of manned airborne LiDAR [15]. Individual tree detection (ITD) accuracy was also relatively high [15]. However, UAS-LiDAR stem density estimation was less accurate than airborne LiDAR due to weaker penetration, large incidence angles and the Ginkgo trees’ unique properties [15]. Jaakkola et al. (2017) used a UAS with the Puck LITE and random forest (RF) regression to estimate DBH with comparable accuracy to terrestrial laser scanning (TLS) and photogrammetric point clouds [16]. Basal area, stem volume and individual tree biomass estimates were also obtained with root mean square errors (RMSEs) adequate for tree-level field measurements [16]. However, direct measurement of DBH from the point cloud was inaccurate due to the Puck LITE’s range accuracy, the inertial measurement unit’s drift and the applied processing algorithms [16].

Quanergy, a company with ties to the automotive industry, released the M8 LiDAR sensor in 2015—half way between when the Puck and the Puck LITE were released (2014 and 2016 respectively) [14]. According to manufacturer specifications, the M8 may have fewer laser beams than Velodyne’s models, but its range, accuracy, weight and price make it a competitive alternative (Table 1) [14]. However, the Quanergy M8 has been relatively untested in the scientific literature with the exception of one study that assessed the M8’s temporal stability and distance measurement quality [17]. Distance measurement error outdoors exceeded the manufacturer-specified accuracy, leading to the conclusion that prolonged heat exposure may negatively impact the M8’s performance [17]. Another study used a mathematical model previously used to predict ALS accuracies and compare that of different UAS laser scanning (ULS) builds, including the M8’s [14]. The predicted horizontal and vertical measurement accuracies of the Velodyne HDL-32E and M8 were comparable; however, it must be noted this model underestimated ULS accuracies observed in other studies [14]. Use of the Puck and Puck LITE on UASs in forestry studies has proven to be an attractive alternative to traditional methodologies such as ALS and TLS. However, there are few studies using these sensor models in wetlands mapping and delineation. To our knowledge, no studies to date have used the Quanergy M8 sensor to collect high resolution LiDAR data for forested wetlands, possibly due to the popularity of the Velodyne models. In this paper, we quantitatively compare point cloud and derived metrics acquired using the Quanergy M8 (estimated at $80,000) and the Velodyne HDL-32E (estimated at $175,000) LiDAR sensors in a mixed palustrine densely forested wetland to determine the relative performance of these sensors when compared with aerial LiDAR. Our overarching research question was whether the Quanergy M8 system could provide comparably high-quality topographic data at half the system acquisition cost.

## 2. Materials and Methods

### 2.1. Study Area Description

**Figure 1:**
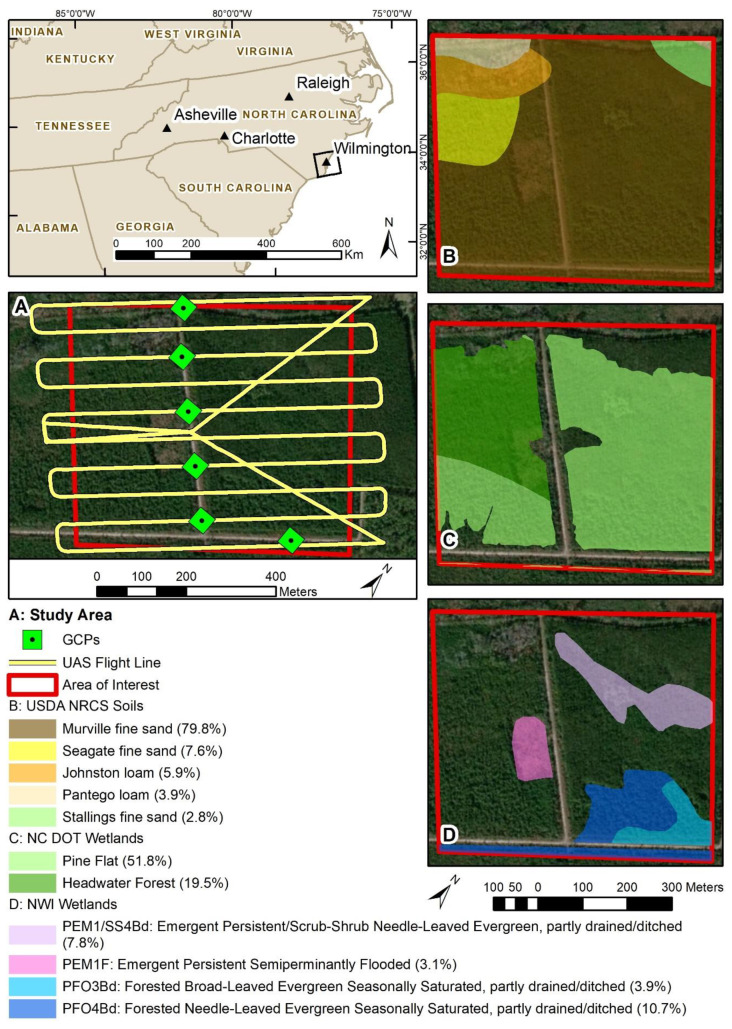
The study area in southeastern North Carolina, USA, dominated by forest cover and showing the UAS flight lines and GCPs (**A**). Additional ancillary data include soils data from the Soil Survey Geographic Database (SSURGO) (**B**), wetlands delineated by the North Carolina Department of Transportation (**C**), and National Wetlands Inventory (**D**).

Our study area is located in southeastern North Carolina, USA and is predominantly forested wetlands, upland pine forest with an understory of extremely dense shrub, and smaller upland grass areas (Figure 1). The degree of re-forestation from previous agricultural land uses varies throughout the study area, with some areas having few trees. Drainage ditches were previously constructed along the old field edges and are typically filled with standing water. Due to continued and necessary development of transportation infrastructure and urban growth across the state of North Carolina, the North Carolina Department of Transportation (NCDOT) continually seeks to reduce development-related impacts to streams and wetlands using mitigation practices. The NCDOT owns several parcels, including this study area, for potential installation of stormwater management devices or best management practices (BMPs) required to secure wetland mitigation credits. This Area of Interest (AOI) is a total of 33.8 hectares, with 24.1 hectares (71% of the total area) consisting of wetlands that were previously mapped by NCDOT using traditional ground survey wetland delineation techniques approved by the COE.

Soils within the study area are dominated by Murville fine sand (79.8%) and three others have much less: Seagate fine sand (7.6%), Johnston loam (5.9%), Pantego loam (3.9%), and Stallings fine sand (2.8%). All are located in very gently sloped terrain from 0 to 2 percent slope (Figure 1, Table 2). Detailed soil descriptions are available through the National Resources Conservation Service Web Soil Survey at https://websoilsurvey.sc.egov.usda.gov/App/HomePage.htm. NWI data within the study area were mapped in 2010 using 1m true color imagery from the National Agriculture Imagery Program (NAIP). More information about these data can be obtained from the Fish and Wildlife Service at https://www.fws.gov/wetlands/Data/SupMapInf/R04Y11P01.pdf. In total, the NWI data comprise 25.5 percent of the study area and are palustrine freshwater wetlands almost equally split between emergent (10.9%) and forested (14.6%). Importantly, most of the wetlands have been ditched or partly drained. Lastly, the NCDOT mapped wetlands within the study area where they delineated two types: 1) headwater forest (6.6 hectares, 19.5%) and 2) pine flat (17.5 hectares, 51.83%). These delineations consisted of an original, verified delineation conducted in 2009–2010, including the tributaries and streams contained in the study area, followed by a second delineation as part of a feasibility document in 2014–2015 that focused only on wetlands.

### 2.2. UAV Platform and LiDAR Sensors

The DJI Matrice 600 Pro (M-600 Pro, manufactured by DJI, Beijing, China) was used to carry both LiDAR payloads used in this study. The M600 Pro is a six-armed rotocoptor with an on-board A3 Pro flight controller and Lightbridge 2 HD transmission system capable of reaching a maximum speed of 65 kph in windless conditions. The M-600 Pro was equipped with six TB48S batteries (LiPo 6S with 22.8 V) for increased flight time. The M-600 Pro was also equipped with a Zenmuse X3 camera that was set up to record video in-flight for safety monitoring purposes only. The M-600 Pro was configured with either the Quanergy M8 or the Velodyne HDL-32E (Table 1). The Velodyne HDL-32E is a 32-laser scanner with a 100 m range (accuracy and precision of 2 cm and 2 mm, respectively). It has a horizontal field of view (FOV) of 360°, vertical FOV of 40°, and is reported to collect up to 1.39 million points per second. The weight of the unit is 1050 g, making it the heavier of the two units we tested. The Quanergy M8 is an eight-laser scanner with a slightly larger range at 150 m (accuracy of 5 cm). It has a 360° horizontal FOV and 20° vertical FOV and weighs 800 g. The Quanergy M8 is reported to collect up to 420,000 points per second using time-of-flight (TOF) depth perception. See Table 1 for a complete listing of sensor specifications.

### 2.3. Data Collection and Initial Processing

On 1 October 2019, the Quanergy M8 and Velodyne HDL-32E LiDAR sensors were used to survey the study area (Figure 1). The Quanergy M8 and VelodyneHDL-32E sensors were provided by LiDARUSA (Hartsell, AL, USA) and eGPS Solutions, Inc. (Norcross, GA, USA), respectively, with flight missions conducted by eGPS Solutions. The same flight mission was used for both flights to ensure direct comparability of the resulting point clouds. Flight missions were created and implemented in DJI Ground Station Pro with a flight altitude of 45 m (150 ft), flight transect spacing of 45 m (150 ft), and flight speed of 12 m/s. Ground control points (GCPs) were used to improve end-product accuracy. Six GCP targets were placed below the planned flight paths and in open view for maximum point density on the target surface. A GeoMax Zenith 35 Pro Global Navigation Satellite System (GNSS) base station (horizontal accuracy 3 mm ± 0.5 ppm RMS, vertical accuracy 5 mm ± 0.5 ppm RMS) and Champion Pro GNSS rover receiver (horizontal accuracy 8 mm ± 1 ppm RMS, vertical accuracy 15 mm ± 1 ppm RMS) were used to establish the six GCP target coordinates with 180 1-s epoch measurements. The base coordinates were derived with the use of the North Carolina Geodetic Survey’s real-time virtual reference station (VRS) network. The final GCP coordinates were derived by taking three redundant measurements with the rover, then averaging those measurements with the base station measurements. Carlson SurvCE data collection software was used to start the base station and record the rover data. Then, a base-rover ultra-high frequency radio method was used to establish the target coordinates and improve the relative accuracy of the targets to the VRS network.

The general data processing methods consisted of: (1) processing raw instrument data to georeferenced the data, remove systematic error, and generate classified point clouds (in LAS format), (2) generation of ground point clouds, (3) assessment of point density and elevation comparisons using tessellation polygons, and (4) comparison of Digital Surface Models (DSMs). The following sections provide detailed descriptions of these methods sequentially.

The telemetry data were post-processed using Novatel Inertial Explorer software and the point cloud was georeferenced using LiDARUSA ScanLook PC software. The raw point cloud was analyzed in the LiDAR module of Global Mapper software to visually assess that the point cloud overlap, depth, and density were sufficient. The unconstrained point cloud was then systematically analyzed in Global Mapper using cross-sections to visually locate and edit any random errors such as double surfaces, excess noise, data gaps, or inconsistencies. ScanLook PC was then used to constrain the point cloud to the GCP targets (average RMSEz of 4.8 ± 2.5 mm for the Quanergy and 6.2 ± 3.7 mm for the Velodyne). Finally, the constrained point cloud was analyzed in Global Mapper to locate systematic or random errors that were previously missed, before export to LAS format (which is a file format designed for the interchange and archiving of LiDAR point cloud data and was developed by the American Society for Photogrammetry and Remote Sensing (ASPRS)).

### 2.4. Point Cloud Post-Processing

**Figure 2:**
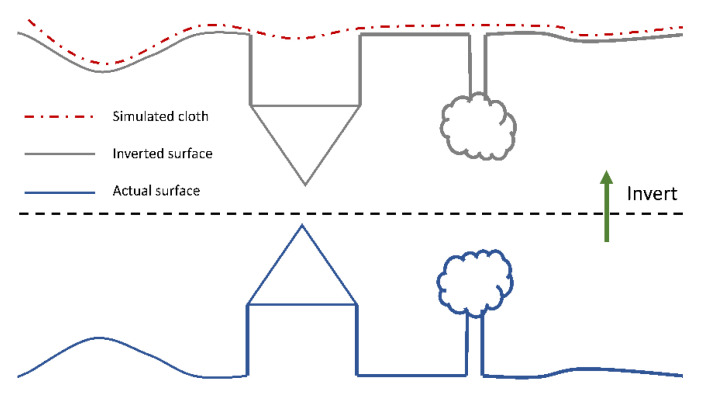
Overview of the Cloth Simulation Filter (CSF). Adapted from Zhang et al., 2016 [18].

Additional post-processing of the LAS files was performed using CloudCompare v2.10.2. CloudCompare is an open source 3D point cloud and mesh processing software available for download at https://www.danielgm.net/cc/. Because Global Mapper outputs the initial point cloud in multiple LAS files, we first merged all LAS files within each flight. The merged file was filtered for ground points using the Cloth Simulation Filter (CSF) plugin [18]. The CSF extracts ground points from discrete return LiDAR point clouds and produces results with accuracies comparable to most state-of-the-art filtering algorithms [18]. Separation of ground and non-ground points is essential in the creation of an accurate digital surface model (DSM). The CSF algorithm is based on a technique called cloth simulation [19]. First, the CSF inverts the original point cloud, and then drapes an imaginary “cloth” over the surface from above (Figure 2). An analysis of the interaction between the nodes of the cloth and the corresponding LiDAR points determines with final shape of the cloth, which is then used as a base to classify the original points into ground or non-ground points.

There are multiple parameters that can be set by the user (a description of available parameters is available in the online Cloth Simulation Filter plugin documentation at https://www.cloudcompare.org/doc/wiki/index.php?title=CSF_(plugin)#General_parameters). The general parameters include Scene and Slope Post Processing for Disconnected Terrain. We opted for the “flat” slope processing, as the study area terrain is generally flat. However, due to the drainage ditches along the dirt roadways, we chose to use Slope Post Processing for Disconnected Terrain to reduce internal constraints that would cause a poor fit of the simulated cloth with the steep slopes of the ditches. Advanced parameter options include Cloth Resolution, Max Iterations, and Classification Threshold. Cloth Resolution refers to the grid size of the cloth and defines the coarseness of the resulting DTM. We defined Cloth Resolution as “1” to ensure a high density of points were captured by the cloth in order to develop a highly resolved ground point cloud file. Other parameters were left as defaults.

### 2.5. Point Cloud Analysis

**Figure 3:**
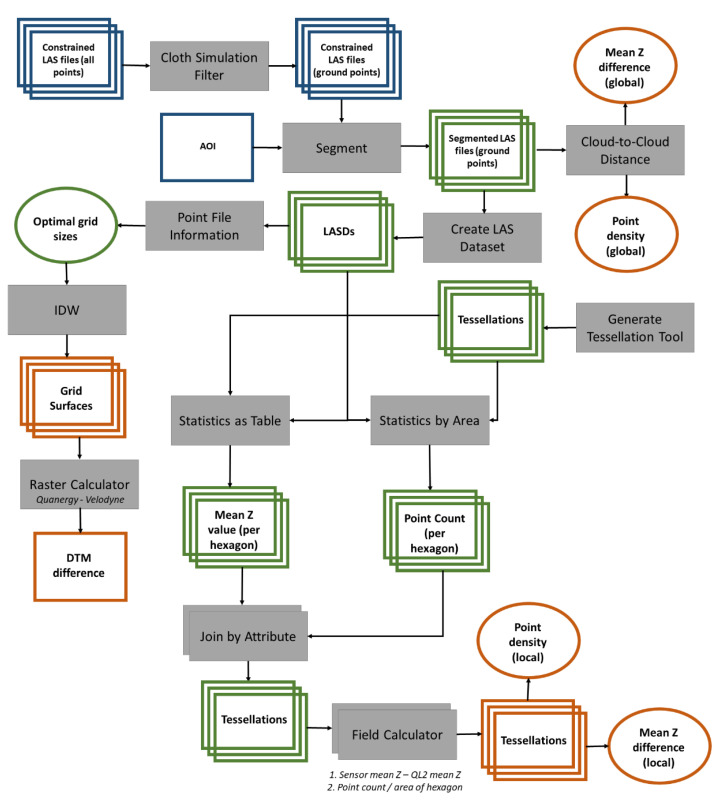
Workflow for tessellation comparative analysis using Quanergy M8, Velodyne HDL-32E, and North Carolina Quality Level 2 (QL2) LiDAR data; grey boxes indicate tools and operations, green squares indicate intermediate outputs from the different operations, dark blue boxes represent inputs including the constrained point clouds, and orange objects indicate final products, with squares indicating spatial data and ovals representing metrics.

Two approaches were used to compare the point cloud sensor data. The first approach was a comparison of the ground point cloud data from each sensor and the second approach compared interpolated DTM surfaces (Figure 3). Ground point clouds from the Quanergy M8 and Velodyne HDL-32E data were compared with the State of North Carolina Quality Level 2 (QL2) LiDAR data available from the Spatial Data Download portal at https://sdd.nc.gov/ [20]. The QL2 LiDAR data were collected in 2014 with a traditional linear aerial sensor at 2 points per meter and uses the coordinate system of NAD83(2011) North Carolina State Plane Feet (US) and the NAV88 Geoid 12A.The data includes multi-return and intensity values. Even though the QL2 is several years older than the LiDAR data collected with the Quanergy and Velodyne, it is the only available LiDAR dataset that is available to use as a reference. The QL2 data were collected under rigorous guidelines by the North Carolina Office of Risk Management in conjunction with NCDOT and other partners, with quality control performed by the North Carolina Geodetic Survey [20]. Accuracy for QL2 data collected in the study area has reported RMSEz for vegetated and non-vegetated areas at 9.0 cm and 6.9 cm, respectively.

We first computed global statistics for the ground point clouds in CloudCompare (Figure 3). A polygon data layer was created using Draw Polyline to define the area of interest (AOI) that best included the areas of maximum point density in both the Quanergy and Velodyne datasets while excluding areas along the edges where scattering from the sensors had occurred. Using Segment, with the AOI and ground clouds as inputs, we created new LAS files that excluded the low-density areas. Then, global point density and mean Z differences were computed between the QL2 ground point cloud to each Quanergy and Velodyne ground point clouds using the Cloud-to-Cloud Distance tool. This tool compares two point clouds and requires the user to assign the roles including “Compared” and “Reference”, where the “Reference” cloud should be the one with the widest extent and highest density. In this case, we set “Reference” as the respective sensor (Quanergy or Velodyne) and “Compared” as QL2 because it had the lower density. We also chose “Split XYZ Component” in order to calculate the mean Z difference with QL2. We applied the Local Modeling strategy, which is based on the least-squares best fitting plane that goes through the nearest point and its neighbors. Local models are sorted by increasing ’fidelity’ to the local geometry, and also by increasing computation time. Specifically, the Quadric (formerly called ’Height function’) model uses a quadratic function (six parameters, Equation (1)) for computing a local model around the nearest point which approximates the real surface and derives a better estimation of the ’real’ distance. We used a radius of 5.8 m (~19 ft) that was automatically determined by CloudCompare based on the less dense cloud (i.e., the QL2 cloud). It is recommended to use the quadratic model because it is more versatile and better represents smooth or curvy surfaces. In this case, we only used the plane normal to choose the dimension for ’Z’. Additional details on tool documentation and parameterization can be found at https://www.cloudcompare.org/doc/wiki/index.php?title=Cloud-to-Cloud_Distance.

Z = a.X^2^ + b.X + c.XY + d.Y + e.Y^2^ + f (1)

The AOI shapefile that was created in CloudCompare was brought into ArcMap. LAS datasets (LASDs) were created from the LAS files for each the Quanergy, Velodyne, and QL2. Using the AOI, the Quanergy, Velodyne, and QL2 ground points were extracted and then compared using local statistics.

To perform a local assessment of the point cloud data from each sensor, we tested several aggregation sampling schemes across the study area. A variety of methods can be used to objectively and independently segment a study area for the purpose of comparing point data through time and space. For example, many studies use stratified sampling approaches, such as linear transects, to compare locations to one another, but the main drawback to this approach is the location of the linear transect could leave out important characteristics in the landscape [21]. Alternatively, dividing space into equal areas, such as fishnet or tessellation, results in all areas being assessed and makes them comparable. Research has shown that sampling through fishnets or tessellations (areas) is preferable to transect lines if the study area can be sampled this way [22]. When aggregating point data to fishnet (squares or rectangles) or tessellation (squares, rectangles, triangles, or hexagons) polygons, the next decision is what shape and size should be used? The size, shape and orientation of the tessellation should be representative of features in the study area. Therefore, in this study area where the terrain is dominated by relatively flat pine forests and circular wetlands, we performed testing and decided on using hexagons since they best represented the area [23]. Using the Generate Tessellation tool in ArcGIS, we compared several hexagon sizes (5 m^2^, 10 m^2^, 15 m^2^, 20 m^2^, and 25 m^2^). Next, we used the ArcGIS tool LAS Point Statistics by Area tool to calculate the minimum, maximum, mean, and standard deviation of elevation and the total point count within each hexagon polygon. Point density was calculated by dividing the point count by area. Spatial sampling across the study area using a hexagon tessellation enabled an objective and unbiased approach to comparing the ground point data from each sensor [22]. We examined differences in elevation and point density in a spatially explicit manner by comparing local variations between sensors and at varying hexagon sizes. Within each tessellation file, the Field Calculator was used to calculate the difference in sensor mean Z relative to QL2 using Equation (2) and difference between sensors using Equation (3).

(2) $$\mathrm{mean}\;Z\;\mathrm{QL}2\;\mathrm{difference}=|Z_{sensor}-Z_{QL2}|$$

(3) $$\mathrm{mean}\;Z\;\mathrm{sensor}\;\mathrm{difference}=|Z_{Velodyne}-Z_{Quanergy}|$$

### 2.6. DTM Analysis

To compare the terrain for each sensor, the ArcGIS Point File Information tool was run for each LASD. This tool uses point binning for improved point spacing calculation and can be used to determine an appropriate spatial resolution for generating terrain rasters from point clouds. We multiplied the point spacing for each file by four and rounded up to the nearest whole number (it is recommended to use a minimum resolution four-times greater than the point spacing). The Quanergy LASD was interpolated to a 0.6 m^2^ (2 ft^2^) grid using Inverse Distance Weighted (IDW). We chose binning rather than triangulation and the linear void fill method for additional parameters. We opted for the high spatial resolution (small grid size) to highlight landscape features such as low-lying wetland areas that are most important in this analysis. The Velodyne LASD was interpolated using the same methodology and co-registered to the Quanergy raster. The QL2 raster was generated using the same methods as above, but at a 3 m^2^ (10 ft^2^) spatial resolution given the larger (average 2 points per meter) point spacing. The QL2 raster was subsequently down-sampled to 0.6 m^2^ (2 ft^2^) spatial resolution and also snapped to the Quanergy raster to ensure that spatial resolution was the same between all rasters and that they lined up properly.

To compare the terrain rasters, we first visually compared the DTMs derived from the Quanergy and Velodyne to each other, to the QL2 DTM, and with aerial imagery obtained from the Esri basemaps in ArcGIS. Next, the ArcGIS Raster Calculator tool was used to subtract the Quanergy DTM from the Velodyne DTM (Equation (4)) to quantitatively assess areas where the interpolated surfaces were similar and different between the DTMs.

(4) $$\mathrm{DTM}\;\mathrm{difference}=|DTM_{Velodyne}-DTM_{Quanergy}|$$

## 3. Results

### 3.1. Cloud to Cloud Comparison

The Quanergy sensor collected slightly more ground points than the Velodyne within the AOI (Table 3). The global ground point density was also higher for the Quanergy, collecting close to 10 more ground points per square meter based on the cloud-to-cloud comparison (Table 3). The global mean Z difference referenced to the QL2 based on the cloud-to-cloud comparison was slightly lower for the Quanergy (0.11 m less than the Velodyne), although the standard deviation of the mean Z difference was higher for the Quanergy sensor (Table 3).

### 3.2. Tessellation Comparison

**Figure 4:**
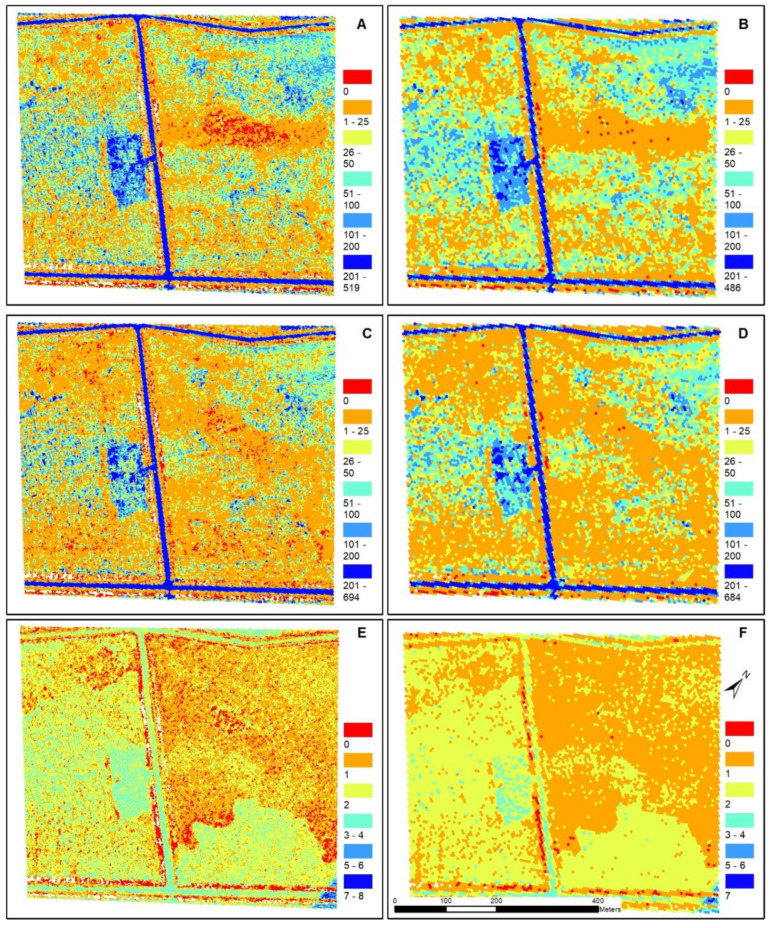
Ground point density (points/m^2^) of two LiDAR UAS sensors, the Quanergy M8 and Velodyne HDL-32E, and North Carolina Quality Level 2 (QL2) LiDAR data: (**A**) Quanergy at 5 m^2^ tessellation, (**B**) Quanergy at 25 m^2^ tessellation, (**C**) Velodyne at 5 m^2^ tessellation, (**D**) Velodyne at 25 m^2^ tessellation, (**E**) QL2 at 5 m^2^ tessellation, and (**F**) QL2 at 25 m^2^ tessellation.

The density of ground points per tessellation varied from one tessellation size to the next. For example, the density of points in the 5 m^2^ tessellation ranged from 0.0 to 518.4 points/m^2^ for the Quanergy and 0.0 to 693.8 points/m^2^ for the Velodyne and the 25 m^2^ tessellation ranged from 0.0 to 485.2 points/m^2^ for the Quanergy and 0.0 to 683.1 points/m^2^ for the Velodyne (Table 4). The results for mean and standard deviation also decreased with increasing tessellation size. The average point density in the 5 m^2^ tessellation was 56.8 ± 78.99 points/m^2^ for the Quanergy and 50.4 ± 95.5 points/m^2^ for the Velodyne and the average points density in the 25 m^2^ tessellation was 55.7 ± 69.6 points/m^2^ for the Quanergy and 49.4 ± 87.3 points/m^2^ for the Velodyne (Table 4). These metrics differed just slightly from the average point density computed using the cloud-to-cloud comparison. The range and average of the QL2 point density were much lower than both UAS sensors we tested (as expected given the reported collection rate of 2 points/m^2^), where the range was 0.0 to 7.4 points/m^2^ in the 5 m^2^ tessellation and 0.0 to 6.5 in the 25 m^2^ tessellation and average was 1.1 ± 0.7 points/m^2^ in the 5 m^2^ tessellation (Table 4). Although the Velodyne had higher maximum point densities compared to the Quanergy, the Quanergy had higher average point densities at all tessellation sizes and the Quanergy sensor also had a higher proportion of polygons with densities above the average (31.8% ± 0.4 compared to 24.8% ± 0.4 for the Velodyne). The tessellation polygons that had densities over 200 points/m^2^ slightly varied by sensor where the Quanergy had an average of 5.1% ± 0.4 and Velodyne had slightly more at 5.5% ± 0.3. The areas with the lowest point density for the Quanergy and Velodyne were in the densest forest cover and the areas of greatest point collection occurred along roads and sparsely vegetated areas (Figure 4).

The average lowest, or minimum, elevation increased with increasing tessellation size (Table 5). For example, the Quanergy was 9.97 m at 5 m^2^ to 10.11 m at 25 m^2^ and Velodyne was 9.95 m at 5 m^2^ to 10.08 m at 25 m^2^. The average minimum elevation of the two UAS sensors were lower than the QL2 LiDAR (differences were all negative) and statistically different from one another (*p* = 0.0399). Conversely, the average highest, or maximum elevation, decreased with increasing tessellation size, were also significantly different (*p* = 0.00001), and differences were all positive which indicates that the two UAS sensors had higher maximum elevations compared with the QL2 LiDAR. Average elevation did not change with increasing tessellation size; however, the sensors were again significantly different (*p* = 0.0004), and the average elevation of the QL2 data was higher than the Quanergy and Velodyne at all tessellation sizes. The UAS sensors had the same average mean Z difference with each other (−0.01) and they had similar differences when compared with the QL2. For example, the Quanergy averaged −0.12 and Velodyne averaged −0.13, indicating the elevation values produced by the UAS sensors were slightly lower than QL2. The average elevation difference between the two UAS sensors, Velodyne to Quanergy, was minor (−0.01 m); however, the difference between the lowest elevation was on average −1.79 (± 0.09 m) and the maximum elevation was 1.52 (± 0.11 m) which indicates that the Velodyne did not capture lowest elevations but it captured higher maximum elevations.

**Figure 5:**
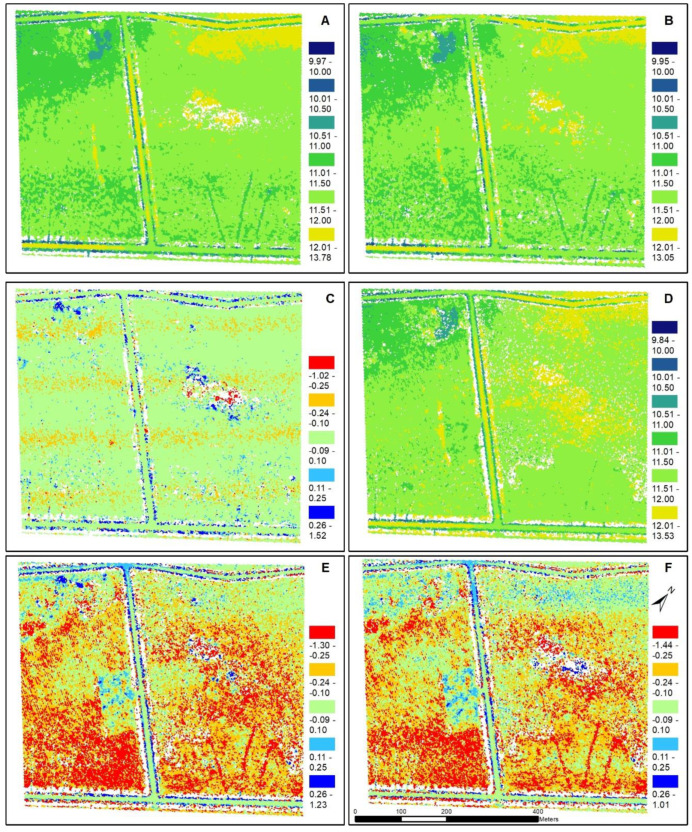
Mean elevation difference (in meters) for the 5 m^2^ tessellation: (**A**) Quanergy M8, (**B**) Velodyne HDL-32E, (**C**) difference between the two UAS LiDAR sensors (Velodyne – Quanergy), (**D**) North Carolina Quality Level 2 (QL2) LiDAR elevation, (**E**) difference between Velodyne and QL2, and (**F**) difference between Quanergy and QL2.

**Figure 6:**
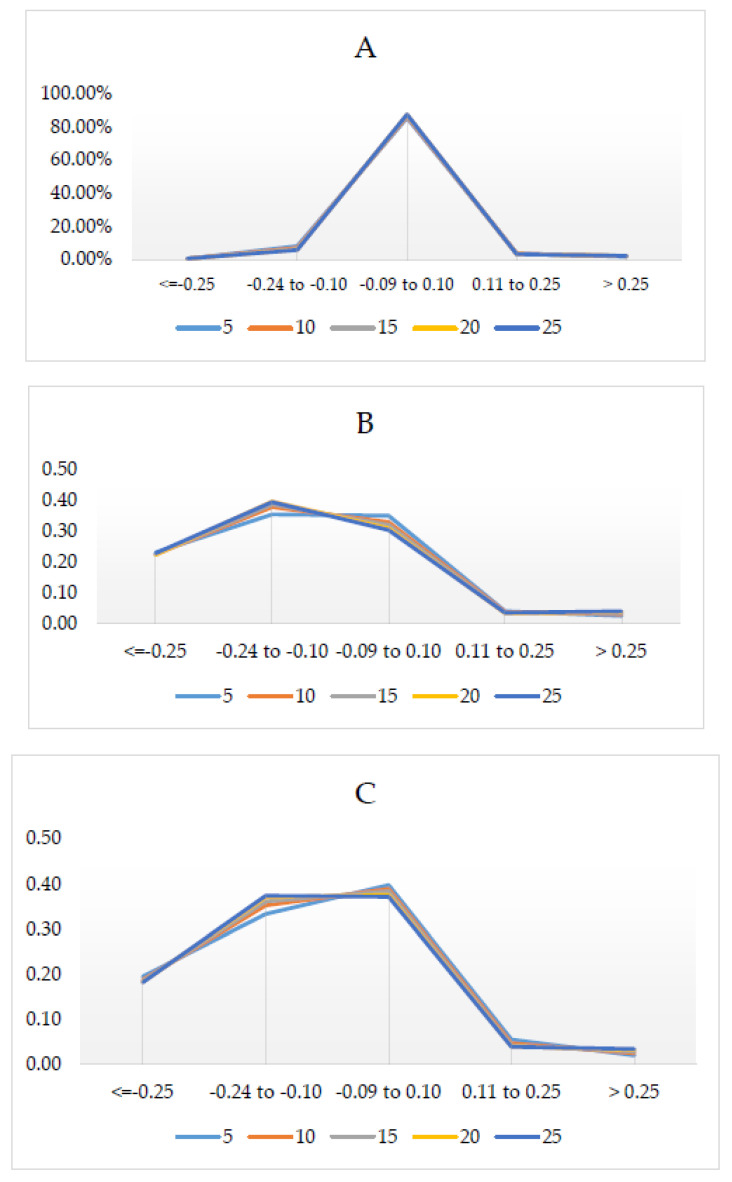
The percentage of area (*Y*-axis) for elevation difference (in meters) (*x*-axis) for each tessellation (5 m^2^ through 25 m^2^) for (**A**) comparison between the Quanergy and Velodyne sensors; (**B**) comparison of Velodyne with the QL2 data, and (**C**) comparison of Quanergy with the QL2 data.

**Figure 7:**
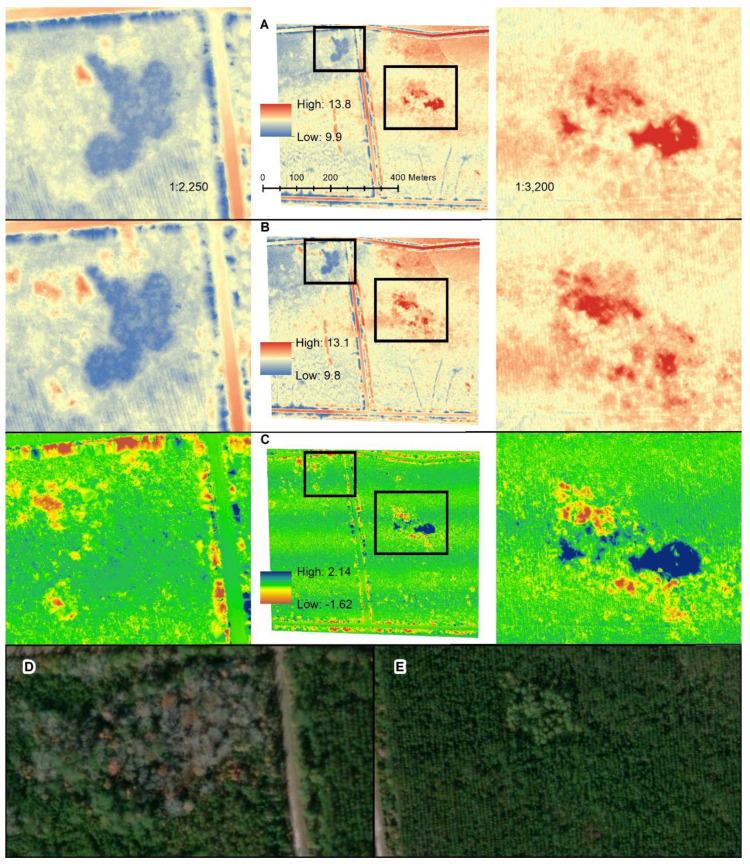
Digital Terrain Models of the Quanergy M8 (**A**) and Velodyne HDL-32E (**B**) LiDAR sensors in low- and high-elevation areas and the difference between them (**C**). Notice the greater (positive) difference are areas where the Velodyne elevation is greater than Quanergy and negative difference is where Velodyne elevation is less than Quanergy. The two highlighted areas are examples where there is little elevation difference and the vegetation canopy is less dense (**D**) and larger differences between the two sensors where there is dense forest canopy (**E**). Imagery shown in (**D**,**E**) are available from Esri online “World imagery” (https://services.arcgisonline.com/ArcGIS/services).

Mean Z differences across the 5 m^2^ tessellation surface are shown in Figure 5. Most (86% ± 0.86) of the difference between Velodyne and Quanergy were minor (−0.09 to 0.10 m) (Figure 5C and Table 6). The ditches on either side of the gravel roads had the greatest differences in elevation. Notably, there was an area/patch in the middle with large elevation differences (positive and negative) which had higher elevations and also had the lowest density of points (Figure 5). Additionally, there was a clear banding/striping in the negative elevation difference (−0. 24 to −0.10 m) (Quanergy had higher elevations than Velodyne). In comparison with the QL2 (Figure 6C,D), the area was dominated by the largest negative difference (−1.30 to −0.25 m), where Velodyne was higher (23% ± 0.33) than Quanergy (19% ± 0.51). Little difference (−0.09 to 0.10 m) was greater for Quanergy (38% ± 1.04) than Velodyne (32% ± 1.84). Lastly, the area was less dominated by UAS elevations greater than QL2; however, the Quanergy slightly out-performed Velodyne where Quanergy had lower area (2.73% ± 0.57) than Velodyne (3.32% ± 0.60). In summary, although the elevations were very similar between Quanergy and Velodyne, both were dominated by negative (lower elevations) difference with QL2 (Figure 7).

### 3.3. DTM Comparison

Elevation in the DTM surfaces ranged from 9.9 to 13.8 m, with an average of 11.6 ± 0.3 m for the Quanergy and 9.8 to 13.1 m with an average of 11.6 ± 0.3 m for the Velodyne (Table 7). The range of the QL2 was 9.9 to 13.4 m with an average of 11.8 ± 0.3 m. Comparison of the DTMs generated from the Quanergy and Velodyne are shown in Figure 7. Visual comparisons of the DTMs generated from the Quanergy and Velodyne are shown in Figure 7 which highlights two areas of low and high elevation difference between the two sensors, respectively, located in the center of our study area. The areas of low difference in DTMs observed between the two sensors present overall little elevation differences and are covered by sparse vegetation while the converse is true where the canopy cover is very dense. The largest difference between the two sensors was observed in the part of the study area with the densest stand of trees (Figure 7C and insets to the right) where LiDAR penetration was the lowest, especially for the Quanergy. Noticeably, there are marked areas of high difference between the DTMs resulting from the two sensors along the ditches immediately adjacent to roads. The vegetation on either side of the road overlying these ditches is unusually dense, pointing to the same issue with cover penetration in dense canopies highlighted above.

## 4. Discussion

As previously stated, our main goal was to ascertain the efficacy of using a lower cost UAS-borne LiDAR sensor to create high-resolution ground products in densely forested, palustrine coastal wetlands. To that effect, we quantitatively compare point cloud and derived topographic metrics acquired using the Quanergy M8 (estimated at $80,000) and the Velodyne HDL-32E (estimated at $175,000) LiDAR sensors to determine the relative performance of these sensors in terms of point cloud and terrain metrics when compared with traditional airborne LiDAR. Overall, we showed that the less expensive Quanergy sensor collected slightly more ground points than the Velodyne (with close to 10 more ground points per square meter based on the global cloud-to-cloud comparison). Similarly, when looking at the global mean difference in elevations (mean Z difference) resulting from the two sensors relative to the QL2 based on the cloud-to-cloud comparisons, we showed that the Quanergy had a smaller relative difference (0.11 m less than the Velodyne), even though the standard deviation of the mean Z difference was higher for the Quanergy sensor. This finding may be partially explained by the 50% higher range of the Quanergy (150 m vs. 100 m for the Velodyne) and lower vertical field of view given the sensors were flown at the exact same altitude, speed, and look angles. This was similarly found by recent research in an urban environment that showed that, when flying at low speeds, the Quanergy M8 is able to achieve significantly higher point density given its smaller horizontal angular resolution [24]. This may also be related to the Quanergy’s accuracy, temporal stability, and distance measurement quality [14,17] despite the lower laser beam count.

Secondly, we assessed the differences in ground point densities between the sensors at a local scale and implemented an iterative area-based random sampling design in order to ensure comparability irrespective of location and point cloud density. In comparing five hexagon sizes ranging incrementally from 5 m^2^ to 25 m^2^, we showed that, similarly to the cloud-to-cloud comparison, the Quanergy generally had slightly higher average point density for all tessellation sizes. This result may again be related to the slightly higher range of the Quanergy despite the much lower number of lasers relative to the Velodyne, as well as its smaller horizontal angular resolution. In addition, given that the focus of our work was to test the performance of the sensors in densely vegetated coastal wetlands, we furthermore computed the average point density at the 5 m^2^ tessellation size for NWI classified wetland types vs. non-wetland covers in our study area (refer back to Figure 1 for the wetland types and Table 8). In comparing the mean point density by NWI class, the average point density for Velodyne was lower than Quanergy for all wetland types and the Quanergy and Velodyne were statistically different (*p* = 0.031679) (Table 8). Conversely, the average point density in wetland areas was not statistically different from the non-wetland areas for both the Quanergy (*p* = 0.112033) and Velodyne (*p* = 0.060544) and they both had equally high standard deviations, indicating that both are prone to errors, especially in the non-wetland/road and ditch areas where the differences between both sensors to the airborne LiDAR and between each other are highest (Figure 7).

When comparing the relative differences between two tested sensors and the QL2 LiDAR-derived DTMs, the highest relative differences were observed in heavily forested areas that typically have lower point density and greater differences in elevation. This issue is common in studies that undertake terrain mapping in dense canopy covers, as is the case with headwater forests that are scrub/shrub dominated and needle-leaved evergreen types [3,7,8]. A high relative difference in DTMs between the sensors was also observed, conversely, in features such as ditches that are smaller and linear and characterized by scrubby, dense vegetation cover. Overall, our findings indicate that the less expensive and lighter payload sensor outperforms the more expensive LiDAR sensor in deriving high-resolution, high-accuracy ground elevation measurements under a range of canopy cover densities. In this study, we only focused on derived ground metrics given the importance of fine variations in topography in the hydrologic functioning, mapping and classification of complex, palustrine wetland systems and we do not report in the performance of the two sensors in classifying digital surface metrics resulting from the collected point clouds. Another potential limitation of this study is that, due to time, resource limitations and our primary focus on acquiring the best possible data for the densely forested type of wetland we surveyed, we only performed these nearly concomitant flights at 45 m AGL (considered ideal for mapping thick canopies) and did not experiment with other flight altitudes. Future surveys at multiple wetland sites across coastal North Carolina will potentially provide opportunities to test multiple flight altitudes.

## 5. Conclusions

In ascertaining the efficacy of collection and resulting terrain features in densely forested palustrine wetland ecosystems collected with two LiDAR sensors mounted on a commercial octocopter, we show that the approaches and methods used to assess LiDAR data are useful, comparable and transferable. The two UAS sensors produced very similar results when assessed against one another and the most recent statewide airborne LiDAR data. We quantitatively assessed these differences in a spatially explicit manner in terms of resulting point densities globally for the study area, as well as locally at varying hexagon sizes. Interestingly, when accounting for wetland types, the average ground point density obtained from the Quanergy was statistically higher than that from the Velodyne sensor. Finally, we computed differences in DTMs derived from both sensors to one another and QL2-derived DTM to show that, overall, the two sensors performed very similarly despite the difference in acquisition price point This research highlights that mapping topography in heavily forested, high-canopy areas that are underlain by wetlands presents significant challenges even when using state-of-the-art active remote sensing platforms and underscores the need for further research and development into more affordable and versatile LiDAR platforms. Finally, despite the relatively high accuracies achieved by both tested LiDAR sensors relative to the statewide QL2 LiDAR, we recommend that thorough field verification be an integral component of mission planning and implementation to assist with terrain processing.

## Figures and Tables

**Table 1:** UAS-mounted LiDAR scanners and specifications, including the Velodyne HDL-32E and Quanergy M8 sensors used in this study; the Velodyne Puck information is included for comparison purposes only (adapted from Pilarska et al. 2016 [14]).

	Velodyne HDL-32E	Quanergy M8	Velodyne VLP-16/Puck
Range [m]	100	150	100
Lasers	32	8	16
Horizontal FOV	360°	360°	360°
Angular resolution	0.16°–0.4°	0.03°–0.2°	0.1°–0.4°
Vertical FOV	40°	20°	30°
Angle between beams	1.33°	2.5°	2°
Range accuracy	2 cm	5 cm	3 cm
Range precision	2 mm	-	2 mm
Dimensions [mm]	85 × 144	97 × 87	103 × 72
Weight	1050 g	800 g	830 g

**Table 2:** Description, area (hectares) and percent of SSURGO soil units within Area of Interest (AOI).

Soil Type and Characteristic	Hectares	Percent
Murville fine sand, 0–2% slope	26.968	79.79
Seagate fine sand, 0–1% slope	2.582	7.64
Johnston loam, 0–2% slope	1.992	5.89
Pantego loam, 0–1% slope	1.311	3.88
Stallings fine sand, 0–2% slope	0.945	2.80

**Table 3:** Basic statistics for cloud-to-cloud comparison performed in CloudCompare using Quanergy M8, Velodyne HDL-32E, and North Carolina Quality Level 2 (QL2) LiDAR datasets. Metrics include the total number of ground points within the area of interest (AOI), point density (calculated by dividing the total points by the area of the AOI), mean Z difference to QL2, and standard deviation (StDev) of mean Z difference to QL2.

Sensor	Total Ground Points	Point Density (pts/m^2^)	Mean Z Difference to QL2	StDev of Mean Z Difference to QL2
Quanergy	18,884,813	55.88	1.34 m	0.93 m
Velodyne	16,773,361	46.63	1.45 m	0.87 m
QL2	365,131	1.08	N/A	N/A

**Table 4:** Point density (points/m^2^) statistics for each sensor (Quanergy, Velodyne and QL2 LiDAR) and tessellation size.

Sensor	Statistic	5 m^2^	10 m^2^	15 m^2^	20 m^2^	25 m^2^
Quanergy	Minimum	0	0	0	0	0
	Maximum	518.4	499.9	494.3	497.9	485.2
	Average	56.8	56.2	55.9	55.8	55.7
	Standard Deviation	78.99	75.1	72.8	70.99	69.6
	Percent Above Average	31.20%	31.60%	31.80%	32.20%	32.00%
Velodyne	Percent Above 200 points/m^2^	5.70%	5.10%	5.00%	4.70%	4.70%
	Minimum	0	0	0	0	0
	Maximum	693.4	693.4	684.5	688.8	683.1
	Average	50.4	49.94	49.7	49.5	49.4
	Standard Deviation	97.5	93.4	90.9	88.8	87.3
	Percent Above Average	24.30%	24.60%	24.90%	25.30%	25.10%
QL2	Percent Above 200 points/m^2^	6.00%	5.60%	5.50%	5.30%	5.30%
	Minimum	0	0	0	0	0
	Maximum	7.4	6.7	6.7	6.7	6.5
	Average	1.1	1.1	1.1	1.1	1.1
	Standard Deviation	0.7	0.6	0.6	0.6	0.6

**Table 5:** Mean elevation (in meters) statistics for each sensor (Quanergy, Velodyne and QL2 LiDAR), tessellation size, and differences between them.

Sensor	Statistic	5 m^2^	10 m^2^	15 m^2^	20 m^2^	25 m^2^	Average	Std. Dev.
Quanergy	Minimum	9.97	9.98	9.98	10.05	10.11	10.02	0.06
	Maximum	13.78	13.78	13.66	13.62	13.71	13.71	0.07
	Average	11.64	11.64	11.64	11.64	11.64	11.64	0.00
	Standard Deviation	0.30	0.30	0.30	0.30	0.30	0.30	0.00
Velodyne	Minimum	9.95	9.96	9.98	10.05	10.08	10.00	0.06
	Maximum	13.05	13.03	13.04	13.03	13	13.03	0.02
	Average	11.63	11.62	11.62	11.62	11.62	11.62	0.00
	Standard Deviation	0.29	0.29	0.29	0.29	0.29	0.29	0.00
QL2	Minimum	9.84	10.11	10.06	10.14	10.18	10.07	0.13
	Maximum	13.53	13.41	13.36	13.44	13.34	13.42	0.08
	Average	11.76	11.76	11.76	11.76	11.76	11.76	0.00
	Standard Deviation	0.31	0.31	0.30	0.30	0.30	0.30	0.01
Quanergy—QL2	Minimum	−1.48	−1.93	−1.96	−1.99	−1.94	−1.86	0.21
	Maximum	2.04	1.79	1.77	1.64	1.65	1.78	0.16
	Average	−0.11	−0.12	−0.12	−0.12	−0.12	−0.12	0.00
	Standard Deviation	0.19	0.19	0.20	0.20	0.21	0.20	0.01
Velodyne—QL2	Minimum	−1.53	−1.45	−1.74	−2.03	−1.78	−1.71	0.23
	Maximum	1.74	1.49	1.59	1.51	1.39	1.54	0.13
	Average	−0.13	−0.13	−0.13	−0.13	−0.13	−0.13	0.00
	Standard Deviation	0.19	0.19	0.20	0.20	0.21	0.20	0.01
Velodyne—Quanergy	Minimum	−1.91	−1.78	−1.83	−1.68	−1.73	−1.79	0.09
	Maximum	1.52	1.5	1.7	1.45	1.41	1.52	0.11
	Average	−0.01	−0.01	−0.01	−0.01	−0.01	−0.01	0.00
	Standard Deviation	0.11	0.11	0.12	0.12	0.12	0.12	0.01

**Table 6:** Mean elevation difference (in meters) between each sensor (Quanergy, Velodyne and QL2 LiDAR) and tessellation size: percentage of study area by classes represented in Figure 6.

Sensors	Elevation Difference (m)	5 m^2^	10 m^2^	15 m^2^	20 m^2^	25 m^2^	Average	Standard Deviation
Velodyne—Quanergy	≤−0.25	0.64%	0.71%	0.77%	0.70%	0.79%	0.72%	0.06%
	−0.24 to −0.10	8.27%	7.18%	6.56%	6.19%	5.93%	6.83%	0.93%
	−0.09 to 0.10	85.34%	86.16%	86.77%	87.25%	87.45%	86.59%	0.86%
	0.11 to 0.25	3.83%	3.68%	3.44%	3.45%	3.40%	3.56%	0.19%
	>0.25	1.93%	2.27%	2.46%	2.40%	2.44%	2.30%	0.22%
Velodyne—QL2	≤−0.25	23.09%	22.47%	22.40%	22.29%	22.81%	22.61%	0.33%
	−0.24 to −0.10	35.44%	37.80%	38.67%	39.64%	39.43%	38.20%	1.70%
	−0.09 to 0.10	35.00%	32.94%	32.03%	30.94%	30.32%	32.25%	1.84%
	0.11 to 0.25	3.99%	3.78%	3.54%	3.33%	3.47%	3.62%	0.26%
	>0.25	2.49%	3.00%	3.36%	3.79%	3.96%	3.32%	0.60%
Quanergy—QL2	≤−0.25	19.46%	18.76%	18.49%	18.27%	18.20%	18.63%	0.51%
	−0.24 to −0.10	33.36%	35.26%	36.17%	37.05%	37.40%	35.85%	1.62%
	−0.09 to 0.10	39.80%	38.94%	38.42%	37.61%	37.20%	38.39%	1.04%
	0.11 to 0.25	5.45%	4.62%	4.16%	3.93%	3.84%	4.40%	0.66%
	>0.25	1.94%	2.42%	2.76%	3.14%	3.37%	2.73%	0.57%

**Table 7:** Digital terrain model (DTM) elevation (m) statistics from Quanergy M8, Velodyne HD32-L, and QL2 LiDAR data.

Sensor	Minimum	Maximum	Average	Standard Deviation
Quanergy	9.9	13.8	11.6	0.3
Velodyne	9.8	13.1	11.6	0.3
QL2	9.9	13.4	11.8	0.3

**Table 8:** Mean point density statistics for wetland vs. non-wetland NWI delineations for the 5 m^2^ tessellation size.

NWI Code	Area (Hectares)	Quanergy		Velodyne	
		Density (Points/m^2^)	Standard Dev	Density (Points/m^2^)	Standard Dev
PEM1/SS4Bd	2.61	35.75	43.24	34.71	44.86
PEM1F	1.03	142.99	90.60	119.51	98.32
PFO3Bd	1.27	26.96	34.35	17.89	32.42
PFO4Bd	3.35	40.72	61.29	32.14	66.35
Non-Wetland	24.64	59.59	83.02	53.86	105.88
Total Area:	32.91				
